# Association of Serum Interleukin-17 Levels With Vitiligo: A Pilot Cross-Sectional Comparative Study

**DOI:** 10.7759/cureus.112983

**Published:** 2026-07-19

**Authors:** Towfiq Ahmad, Munir Rashid, Mohammad Abul Kalam Azzad, ATM Asaduzzaman, Mohammad Abul Kalam Azad, Arjun Saha, Md. Abir Hassan, Tanzina Begum Chowdhury, Wasi Deen Ahmed, Marufa Shahrin

**Affiliations:** 1 Dermatology and Venereology, Bangladesh Medical University, Dhaka, BGD; 2 Rheumatology, Bangladesh Medical University, Dhaka, BGD

**Keywords:** cytokine, il-17, interleukin-17, vasi, vasi score, vitiligo, vitiligo area scoring index

## Abstract

Background: Vitiligo is the most common acquired depigmenting disorder characterized by immune-mediated destruction of epidermal melanocytes. Interleukin-17 (IL-17), a pro-inflammatory cytokine involved in immune responses, has been reported to be elevated in patients with vitiligo; however, its role in disease pathogenesis remains uncertain. This pilot study aimed to evaluate the association between serum IL-17 levels and vitiligo.

Objectives: To explore the relationship between serum IL-17 levels and vitiligo.

Methods: Following institutional ethical approval, this pilot cross-sectional comparative study was conducted in the Department of Dermatology and Venereology, Bangladesh Medical University, Dhaka. The study included 25 patients with stable vitiligo of any clinical type, diagnosed clinically and confirmed by Wood's lamp examination, and 25 age- and sex-matched healthy controls. Disease severity was assessed using the Vitiligo Area Scoring Index (VASI), and serum IL-17 concentrations were measured by enzyme-linked immunosorbent assay (ELISA).

Results: Age and sex distribution did not differ significantly between the vitiligo patients and the control group (P > 0.05). However, serum IL-17 levels were notably higher in vitiligo patients than in healthy controls, at 41.3 ± 35.9 pg/mL versus 17.34 ± 13.8 pg/mL (P = 0.003).

There was a strong positive correlation between IL-17 levels and disease severity, as measured by the VASI score (Rho = 0.790, P < 0.001). However, no association was detected with factors such as age, disease duration, or age at onset. Through receiver operating characteristic (ROC) curve analysis, a threshold of 10.9 pg/mL was established, showing 96% sensitivity and 70% accuracy. Higher IL-17 levels were significantly associated with an increased risk of vitiligo (OR = 18, P < 0.001).

Conclusion: Serum IL-17 levels were notably higher in vitiligo patients, correlated with disease severity, and could serve as a sensitive diagnostic marker, with levels ≥10.9 pg/mL strongly associated with the condition.

## Introduction

Vitiligo is a chronic acquired depigmenting disorder characterized by the progressive loss of functional melanocytes, resulting in well-demarcated depigmented macules and patches affecting the skin and, occasionally, mucosal surfaces [1,2]. It is widely recognized as an autoimmune disease in which autoreactive cytotoxic CD8⁺ T lymphocytes selectively target and destroy melanocytes through immune-mediated mechanisms [3]. Interferon-γ (IFN-γ)-dependent signaling pathways play a pivotal role in disease initiation and progression by promoting the recruitment, activation, and persistence of melanocyte-specific autoreactive T cells within affected skin [3,4]. Increasing evidence indicates that dysregulation of inflammatory cytokines, including interleukin-17 (IL-17), tumor necrosis factor-α (TNF-α), and other pro-inflammatory mediators, contributes substantially to melanocyte injury and disease progression [5,6]. Furthermore, impaired immune tolerance, particularly dysfunction of regulatory T cells, may facilitate uncontrolled autoimmune responses against melanocytes [7]. Systemic inflammatory alterations and immune dysregulation have also been reported in patients with vitiligo, supporting the concept that vitiligo represents a broader immune-mediated disorder rather than an isolated cutaneous condition [8].

The immunopathogenesis of vitiligo involves complex interactions between innate and adaptive immune responses. Oxidative stress in melanocytes is considered an important initiating event, leading to cellular damage and the activation of innate immune pathways that promote melanocyte-specific CD8⁺ T-cell responses [3,9]. Activated melanocyte-specific CD8⁺ T cells migrate to the skin and induce melanocyte apoptosis via IFN-γ-mediated immune mechanisms, leading to progressive depigmentation [3,4]. Persistent inflammation is maintained by cytokine dysregulation and defective immune regulatory mechanisms, contributing to disease chronicity and progression [5,7]. In addition, psychological stress and neuroendocrine-immune interactions may influence inflammatory pathways and immune cell profiles, potentially affecting disease activity and progression [10].

IL-17 is an important pro-inflammatory cytokine implicated in autoimmune and inflammatory skin diseases. Primarily produced by T helper 17 (Th17) cells, IL-17 promotes recruitment and activation of inflammatory cells and stimulates the production of additional cytokines, including TNF-α and IL-6, thereby amplifying inflammatory responses [11]. Increased IL-17 expression in serum and lesional skin has been demonstrated in patients with vitiligo, suggesting its involvement in disease pathogenesis by amplifying inflammatory cascades and immune-mediated melanocyte destruction [12,13]. IL-17 may further contribute to vitiligo progression by interacting with IFN-γ-driven pathways and promoting cytotoxic T-cell-mediated immune responses against melanocytes [3,11,14]. Recent clinical studies have also reported elevated IL-17 levels in patients with nonsegmental vitiligo and suggested an association with disease activity and severity [12,13].

Although substantial progress has been made in understanding the immune mechanisms underlying vitiligo, the clinical significance of circulating IL-17 levels and their relationship with disease severity remain incompletely understood. Previous studies have demonstrated increased IL-17 concentrations in patients with vitiligo; however, the association with disease severity varies across populations and study designs [12-14]. Moreover, evidence from South Asian populations, particularly Bangladesh, remains limited. Therefore, this study aimed to compare serum IL-17 levels between patients with vitiligo and healthy controls and to evaluate the association between serum IL-17 concentrations and disease severity among Bangladeshi patients with vitiligo.

## Materials and methods

Study design and setting

This pilot, cross-sectional, comparative study was conducted at the Department of Dermatology and Venereology, Bangladesh Medical University (BMU; formerly Bangabandhu Sheikh Mujib Medical University), Dhaka, Bangladesh, from May 2023 to March 2025.

Participants

Patients with vitiligo (group A) and healthy controls (group B) were enrolled. Healthy controls were frequency-matched to patients with vitiligo by age and sex and were recruited from patient attendants, healthcare professionals, and hospital employees who had no history of vitiligo, autoimmune disease, or chronic inflammatory disorders. Participants were recruited consecutively throughout the study period.

Eligibility criteria

Individuals of any age or sex with clinically diagnosed stable vitiligo of any clinical subtype were eligible for inclusion in the patient group. Stable vitiligo was defined as the absence of new lesions, enlargement of existing lesions, or spontaneous repigmentation during the preceding six months. The diagnosis of vitiligo was established clinically by a consultant dermatologist and, when required, confirmed by Wood's lamp examination to identify subclinical lesions or better delineate lesion margins.

Healthy controls were individuals without vitiligo or a history of autoimmune or chronic inflammatory disorders.

Participants with autoimmune or chronic inflammatory diseases other than vitiligo, active infection, malignancy, pregnancy, or lactation were excluded. Individuals who had received topical or systemic treatment for vitiligo, immunosuppressive or immunomodulatory therapy, or phototherapy within the preceding three months were also excluded to minimize potential confounding effects on serum IL-17 concentrations.

Sample size

As this was an exploratory pilot study, the sample size was determined pragmatically based on feasibility and previous evidence regarding the role of IL-17 in vitiligo pathogenesis [15]. A total of 50 participants were enrolled, including 25 patients with vitiligo and 25 healthy controls frequency-matched for age and sex. Consecutive sampling was used throughout the study period. No formal hypothesis-driven sample size calculation or statistical power analysis was performed.

Study variables

The primary outcome was serum IL-17 concentration (pg/mL). Secondary outcomes included disease severity assessed using the Vitiligo Area Scoring Index (VASI) and the Facial Vitiligo Area Scoring Index (F-VASI) [16,17]. The primary exposure variable was vitiligo status (patient versus healthy control). Among patients with vitiligo, the association between serum IL-17 concentration and disease severity was evaluated.

Clinical evaluation and data collection

Demographic and clinical data were collected using a structured questionnaire. Recorded variables included age, sex, age at disease onset, disease duration, family history of vitiligo, previous treatment history, and clinical subtype.

Disease severity was assessed at baseline using the VASI. This validated clinical instrument estimates the extent of depigmentation according to body surface area involvement and the degree of residual pigmentation [16]. Facial involvement was assessed using the F-VASI, a validated modification specifically designed to evaluate facial vitiligo [17].

For exploratory subgroup analysis, patients were categorized into three VASI severity groups (<5, 5-10, and >10) to compare serum IL-17 concentrations across disease severity categories.

Laboratory analysis

Venous blood samples were collected under aseptic conditions. Serum was separated by centrifugation and stored at −20°C until analysis. Serum IL-17 concentrations were measured using a commercially available enzyme-linked immunosorbent assay (ELISA) kit according to the manufacturer's instructions. Laboratory personnel performing IL-17 measurements were blinded to participants' case-control status to minimize measurement bias. Serum IL-17 concentrations were expressed as pg/mL.

Bias control

Selection bias was minimized through consecutive recruitment and frequency matching of cases and controls according to age and sex. Measurement bias was minimized by blinding laboratory personnel to participants' case-control status during serum IL-17 assessment.

Statistical analysis

Data were analyzed using IBM SPSS Statistics for Windows, version 26.0 (IBM Corp., Armonk, NY, USA). Continuous variables were assessed for normality using the Shapiro-Wilk test. Normally distributed variables are presented as mean ± standard deviation (SD), whereas non-normally distributed variables are presented as median (interquartile range (IQR)). Categorical variables are presented as frequencies and percentages.

Comparisons between two independent groups were performed using the independent-samples t-test for normally distributed variables or the Mann-Whitney U test for non-normally distributed variables. Categorical variables were compared using the chi-square test or Fisher's exact test, as appropriate.

Differences in serum IL-17 concentrations across the three VASI severity categories were evaluated using the Kruskal-Wallis test. When statistically significant differences were observed, post hoc pairwise comparisons with appropriate correction for multiple testing were performed.

Correlations between serum IL-17 concentrations and clinical variables, including VASI score, were assessed using Spearman's rank correlation coefficient.

Receiver operating characteristic (ROC) curve analysis was performed to evaluate the diagnostic performance of serum IL-17 concentrations for distinguishing patients with vitiligo from healthy controls. The area under the curve (AUC) and corresponding 95% confidence interval (CI) were calculated. All statistical tests were two-sided, and a p-value <0.05 was considered statistically significant.

Ethical considerations

The study was approved by the Institutional Review Board of Bangladesh Medical University (Approval No. BSMMU/2024/1450). Written informed consent was obtained from all participants or their legal guardians before enrollment. Participant confidentiality and anonymity were maintained throughout the study. The study was conducted in accordance with the ethical principles of the Declaration of Helsinki [18].

Quality control

Standardized procedures were followed for clinical assessment, specimen collection, laboratory analysis, and data management. All study procedures were supervised by the investigators. Data were reviewed regularly for completeness, consistency, and accuracy. Laboratory analyses were performed according to standard operating procedures to ensure the quality and reliability of the study data.

## Results

The study included 50 participants, comprising 25 patients with vitiligo (group A) and 25 healthy controls (group B). The control group was frequency-matched to the vitiligo group for age and sex. Baseline demographic characteristics, including age, sex, and occupation, were comparable between the two groups (all P > 0.05) (Table 1).

**Table 1:** Baseline demographic characteristics of the study participants (N = 50). ᵃ Unpaired t-test. ᵇ Chi-square test. P < 0.05 is considered statistically significant.

Epidemiological profile	Group A (n = 25)	Group B (n = 25)	Test statistic	P-value
Age group (years)	n (%)	n (%)	-	-
≤10	3 (12.0)	2 (8.0)	-	-
11-20	7 (28.0)	8 (32.0)	-	-
21-30	5 (20.0)	7 (28.0)	-	-
31-40	3 (12.0)	2 (8.0)	-	-
>40	7 (28.0)	6 (24.0)	χ² = 0.74	0.946
Mean ± SD	27.9 ± 14.9	26.9 ± 13.5	t = 0.25	0.789ᵃ
Range (min-max)	4-58	5-56	-	-
Sex			χ² = 0.33	0.569ᵇ
Male	10 (40.0)	12 (48.0)	-	-
Female	15 (60.0)	13 (52.0)	-	-
Occupational status			χ² = 7.23	0.204ᵇ
Service	5 (20.0)	9 (36.0)	-	-
Business	2 (8.0)	3 (12.0)	-	-
Student	9 (36.0)	10 (40.0)	-	-
Housewife	7 (28.0)	1 (4.0)	-	-
Fisherman	1 (4.0)	0 (0.0)	-	-
Others	1 (4.0)	2 (8.0)	-	-

**Figure 1:**
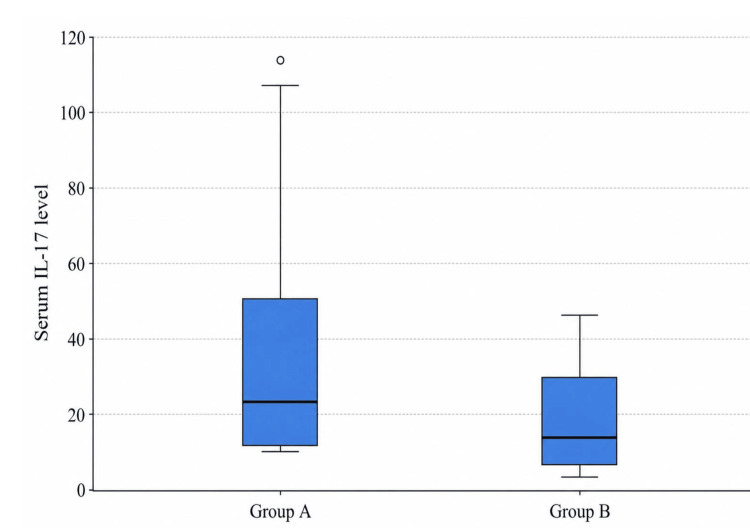
Box plot showing the central tendency of IL-17 levels in group A and group B. Comparison of serum interleukin-17 (IL-17) levels between the study groups. Box-and-whisker plots showing the distribution of serum IL-17 levels in group A and group B. The central line within each box represents the median, the box indicates the interquartile range (IQR; 25th-75th percentiles), and the whiskers represent the minimum and maximum values excluding outliers. The circle denotes an outlier observation. Group A demonstrated higher serum IL-17 levels and greater variability compared with group B.

Serum IL-17 concentrations were significantly higher in patients with vitiligo than in healthy controls (median: 34.10 vs. 12.80 pg/mL; Mann-Whitney U test, P =0.003) (Table 2). Figure 1 illustrates the distribution of serum IL-17 concentrations in both groups, demonstrating higher values and greater variability among patients with vitiligo.

**Table 2:** Serum interleukin-17 (IL-17) concentrations in patients with vitiligo (group A) and healthy controls (group B) (N = 50). Serum interleukin-17 (IL-17) concentrations are presented as mean ± standard deviation (SD), median (range), and interquartile range (IQR). Comparisons between patients with vitiligo and healthy controls were performed using the Mann-Whitney U test. All P-values are two-sided, and a P-value <0.05 was considered statistically significant.

Parameter	Group A (n = 25)	Group B (n = 25)	P-value
Mean ± SD (pg/mL)	41.3 ± 35.9	17.34 ± 13.8	–
Median (range), pg/mL	23.1 (10.50-115.20)	13.3 (3.10-46.30)	0.003
Interquartile range (IQR), pg/mL	11.7-55.9	6.18-29.7	–

Serum IL-17 concentrations differed significantly across VASI categories (Kruskal-Wallis H = 7.15, df = 2, P = 0.028) (Table 3). Patients with a VASI score >10 had the highest serum IL-17 concentration (58.15 ± 43.50 pg/mL), whereas those with VASI scores of 5-10 had the lowest concentration (16.00 ± 6.12 pg/mL). Patients with VASI scores <5 had intermediate serum IL-17 concentrations (31.70 ± 19.28 pg/mL).

**Table 3:** Serum IL-17 levels across VASI categories among patients with vitiligo (group A, n = 25). Data are presented as mean ± standard deviation (SD), median, and range (minimum-maximum). Differences in serum IL-17 levels across VASI categories were analyzed using the Kruskal-Wallis test. A two-sided P-value < 0.05 was considered statistically significant. VASI: Vitiligo Area Scoring Index.

VASI category	n	Mean ± SD (pg/mL)	Median (pg/mL)	Range (min-max)
<5	8	31.70 ± 19.28	32.60	11.60-61.60
5-10	5	16.00 ± 6.12	11.70	11.40-23.10
>10	12	58.15 ± 43.50	43.80	10.50-115.20

Spearman's correlation analysis demonstrated a strong positive correlation between serum IL-17 concentrations and VASI score (ρ = 0.790, P < 0.001). No significant correlations were observed between serum IL-17 concentrations and age (ρ = 0.148, P = 0.480), disease duration (ρ = 0.210, P = 0.314), or age at disease onset (ρ = −0.007, P = 0.972) (Table 4).

**Table 4:** Correlation of serum interleukin-17 (IL-17) concentrations with clinical parameters among patients with vitiligo (group A, n = 25). Correlations between serum interleukin-17 (IL-17) concentrations and clinical parameters were assessed using Spearman's rank correlation coefficient (ρ). Positive values of ρ indicate a direct association, whereas negative values indicate an inverse association. All P-values are two-sided, and statistical significance was defined as P < 0.05. VASI: Vitiligo Area Scoring Index. * Statistically significant (P < 0.05).

Clinical parameter	Spearman's correlation coefficient (ρ)	P-value
Age (years)	0.148	0.480
Disease duration (years)	0.210	0.314
Age at onset (years)	-0.007	0.972
VASI score	0.790	<0.001*

**Figure 2:**
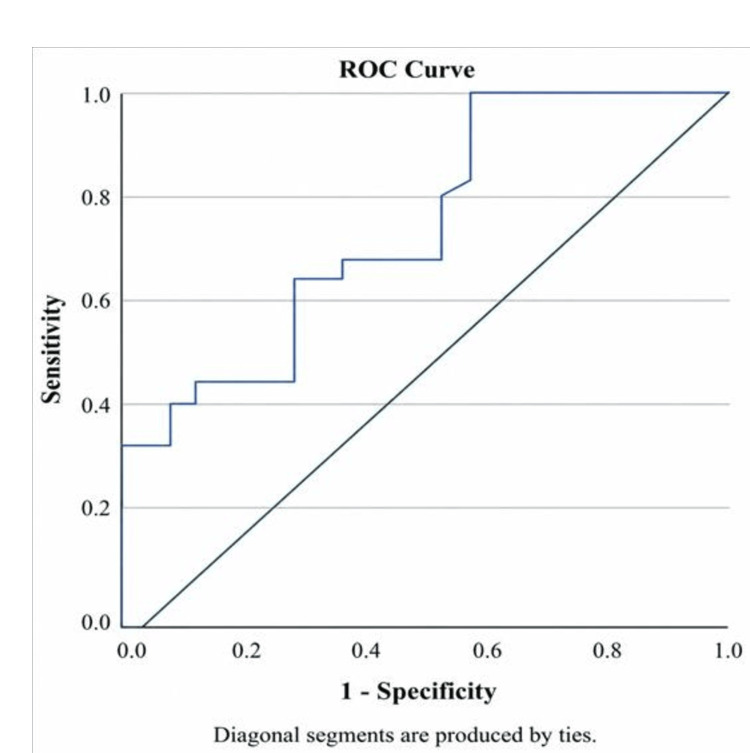
Receiver operating characteristic (ROC) curve analysis used to determine the optimal cut-off value of serum IL-17 for distinguishing vitiligo patients from healthy individuals. The ROC curve demonstrates the discriminatory performance of serum IL-17 concentrations, with an area under the curve (AUC) of 0.745 (95% CI, 0.610-0.880).

ROC curve analysis demonstrated fair discriminatory performance of serum IL-17 for distinguishing patients with vitiligo from healthy controls (AUC = 0.745; 95% CI, 0.610-0.880; P = 0.003) (Figure 2 and Table 5). At the optimal cutoff value of 10.9 pg/mL, serum IL-17 showed a sensitivity of 96.0%, specificity of 44.0%, and an overall diagnostic accuracy of 70%.

**Table 5:** Receiver operating characteristic (ROC) curve analysis of serum IL-17 levels for differentiating vitiligo patients from healthy individuals (N = 50). ROC curve analysis demonstrated that serum IL-17 had fair diagnostic performance in differentiating vitiligo patients from healthy individuals, with an area under the curve of 0.745 (95% CI: 0.610-0.880), which was statistically significant (P < 0.05). At the optimal cut-off value of 10.9, serum IL-17 showed high sensitivity (96.0%) but low specificity (44.0%). The positive and negative predictive values were 63.2% and 91.7%, respectively, with an overall diagnostic accuracy of 70.0%.

	Cut-off value	Sensitivity	Specificity	Positive predictive value	Negative predictive value	Accuracy	Area under the ROC curve	P-value	95% confidence interval (CI)	
									Lower bound	Upper bound
Serum IL-17	10.9	96	44.0	63.2	91.7	70.0	0.745	0.003	0.610	0.880

Serum IL-17 concentrations ≥10.9 pg/mL were significantly associated with the presence of vitiligo (OR = 18.86; 95% CI, 2.20-161.99; Fisher's exact test, P = 0.0019) (Table 6).

**Table 6:** Association of cut-off value of IL-17 between group A and group B (N = 50). ^†^ Fisher's exact test (two-sided).

Serum IL-17 level (pg/mL)	Vitiligo (n = 25), n (%)	Controls (n = 25), n (%)	Odds ratio (95% CI)	P-value^†^
≥10.9	24 (96.0)	14 (56.0)	18.86 (2.20–161.99)	0.0019
<10.9	1 (4.0)	11 (44.0)	–	–

A significantly higher proportion of participants in group A had serum IL-17 levels ≥10.9 pg/mL than in group B (24 (96.0%) vs. 14 (56.0%)). In contrast, serum IL-17 levels <10.9 pg/mL were more common in group B (11 (44.0%) vs. 1 (4.0%)). This difference was statistically significant (Fisher's exact test, P = 0.0019). Participants with serum IL-17 levels ≥10.9 pg/mL had higher odds of having vitiligo (OR = 18.86, 95% CI: 2.20-161.99). However, the wide confidence interval indicates considerable imprecision, likely reflecting the small sample size of this pilot study.

## Discussion

In this pilot cross-sectional study, serum IL-17 concentrations were significantly higher in patients with vitiligo than in healthy controls. They demonstrated a strong positive correlation with disease severity assessed by the VASI. These findings support previous evidence that Th17-mediated immune responses contribute to the immunopathogenesis of vitiligo. IL-17 is involved in chronic inflammatory responses through the activation of immune cells, the induction of pro-inflammatory cytokines, and the amplification of autoimmune pathways implicated in melanocyte destruction [19,20].

The demographic characteristics of the study groups were comparable with respect to age, sex, and occupation, reducing the likelihood of confounding by these variables. Vitiligo can occur at any age, although it is more common during childhood and early adulthood [21]. In the present study, no significant difference in sex distribution was observed between patients with vitiligo and healthy controls, which is consistent with previous reports showing no consistent sex predominance in vitiligo. Occupational status was also comparable between groups. Although occupation itself is not considered a direct cause of vitiligo, environmental exposures and psychological stress associated with certain occupations may contribute to disease initiation or exacerbation through neuroimmune and inflammatory mechanisms [22].

A major finding of this study was the significantly elevated serum IL-17 concentration among patients with vitiligo compared with healthy controls. This finding is consistent with previous studies demonstrating increased serum IL-17 levels in patients with vitiligo and supports the contribution of Th17-mediated inflammation to disease pathogenesis [23-26]. IL-17, primarily secreted by Th17 lymphocytes, promotes inflammatory responses by inducing chemokine production, facilitating recruitment of immune cells, and sustaining chronic inflammation. Experimental and clinical studies suggest that IL-17 may contribute to melanocyte injury through interactions with other inflammatory mediators, particularly IFN-γ and TNF-α, which are central mediators in autoimmune melanocyte destruction [19,20,25,26]. However, IL-17 should be interpreted as one component of a complex cytokine network rather than a single causative factor.

In this study, serum IL-17 concentrations increased progressively across VASI severity categories, and a strong positive correlation was observed between IL-17 levels and VASI score. These findings indicate that higher circulating IL-17 concentrations are associated with greater disease severity. Similar associations between increased IL-17 expression and more extensive or severe vitiligo have been reported previously [23,24,26]. Nevertheless, the cross-sectional design prevents the determination of temporal relationships or causal inference. Elevated IL-17 may represent increased inflammatory activity secondary to extensive disease rather than directly driving disease progression. Furthermore, no significant correlation was observed between serum IL-17 concentration and age, disease duration, or age at disease onset, suggesting that these clinical variables were not major determinants of circulating IL-17 levels in this cohort.

ROC curve analysis demonstrated a moderate discriminative ability of serum IL-17 concentrations in distinguishing patients with vitiligo from healthy controls (AUC = 0.745). Although the selected cutoff value showed high sensitivity, the relatively low specificity limits its application as an independent diagnostic biomarker. Previous studies have similarly suggested that inflammatory cytokines may have greater clinical utility as supportive biomarkers when combined with clinical assessment and other laboratory parameters, rather than as standalone diagnostic tests [19,23].

Serum IL-17 concentrations ≥10.9 pg/mL were significantly associated with the presence of vitiligo (OR = 18.86; 95% CI: 2.20-161.99). However, the wide confidence interval indicates limited precision, likely due to the relatively small sample size of this pilot study. Therefore, this finding should be interpreted cautiously and requires confirmation in larger studies with adequate statistical power.

Overall, this pilot study demonstrates that serum IL-17 concentrations are elevated in patients with vitiligo and are positively associated with disease severity. These findings reinforce the role of Th17-mediated immune pathways in vitiligo pathogenesis and suggest that serum IL-17 may serve as a potential marker of inflammatory activity. However, the moderate diagnostic performance indicates that IL-17 alone is unlikely to replace clinical evaluation. Future multicenter, prospective studies with larger sample sizes, longitudinal follow-up, and comprehensive cytokine profiling are required to validate these findings and clarify the clinical significance of IL-17 as a biomarker and potential therapeutic target in vitiligo.

Limitations

This study has several limitations. The pilot design, relatively small sample size, and single-center setting may limit the generalizability of the findings, while the cross-sectional design precludes causal inference. Potential confounding factors, including vitiligo subtype and metabolic comorbidities, were not evaluated. In addition, only serum IL-17 concentrations were measured, and other inflammatory cytokines involved in the pathogenesis of vitiligo were not assessed. Finally, serum IL-17 concentrations were measured at a single time point; therefore, temporal changes in cytokine levels could not be evaluated. Larger, multicenter, longitudinal studies incorporating comprehensive cytokine profiling are warranted to validate these findings and further clarify the role of IL-17 as a biomarker and potential therapeutic target in vitiligo.

## Conclusions

Overall, the present pilot study suggests that serum IL-17 concentrations are elevated in patients with vitiligo and are positively associated with disease severity. These findings are consistent with previous evidence indicating that Th17-mediated immune responses may contribute to the immunopathogenesis of vitiligo. Although serum IL-17 demonstrated moderate discriminatory performance, it should be considered a supportive biomarker rather than a standalone diagnostic marker. Larger prospective, multicenter studies are needed to validate these findings and further clarify the clinical utility of serum IL-17 as a biomarker in vitiligo.
